# Long-Term Benefits of an Integrated Continuous Glucose Monitoring and Insulin Pump System for Emergency Admissions, Hospitalization, and Metabolic Control in a Cohort of People With Diabetes: Retrospective Cohort Study

**DOI:** 10.2196/46880

**Published:** 2023-08-23

**Authors:** Miguel O'Meara, Juan Camilo Mateus Acuña, Andrea Uribe

**Affiliations:** 1 Fundación Cardioinfantil Universidad del Rosario Programa Diabetes de alta complejidad, Compensar Entidad Promotora de salud Bogotá Colombia; 2 Pontificia Universidad Javeriana Bogotá Colombia; 3 Clínica Los Cobos Medical Center Universidad del Bosque Bogotá Colombia

**Keywords:** automated insulin delivery, continuous glucose monitoring, CGM, glycemic control, hypoglycemia, sensor-augmented insulin pump, type 1 diabetes

## Abstract

**Background:**

There is evidence in the literature that the use of sensor-augmented insulin pumps in patients with high-complexity diabetes improves metabolic control. However, there is no long-term information on clinical outcomes such as hospitalization or admission to the emergency room. This study describes outcomes for metabolic control, incidence of hospitalizations, and emergency room visits in a specific population using this technology.

**Objective:**

We aimed to assess long-term glycemic and clinical outcomes after the use of continuous subcutaneous insulin infusion and continuous glucose monitoring in people with diabetes.

**Methods:**

A retrospective cohort study was carried out in patients with diabetes previously treated with an intensive insulin regimen at a specialized diabetes treatment center who required a sensor-augmented insulin pump due to nonoptimal glycemic control. Glycated hemoglobin, severe hypoglycemic episodes, nonsevere hypoglycemic episodes, perception of hypoglycemia, and the incidence of emergency room visits and hospitalizations before and after treatment were evaluated.

**Results:**

Between January 2013 and August 2020, 74 patients with a median age of 36 (IQR 27-46) years were included in the study with a median 4 (IQR 2-7) years of follow-up. We found a statistically significant reduction in glycated hemoglobin (8.35% vs 7%; *P*<.001), nonsevere hypoglycemic episodes (71/74, 96% vs 62/74, 84%; *P*=.01), emergency room visits (42/73, 58% vs 4/62, 6%; *P*<.001), and hospitalizations (36/72, 50% vs 10/72, 14%; *P*<.001) after use of continuous subcutaneous insulin infusion.

**Conclusions:**

The use of a sensor-augmented insulin pump associated with a strict follow-up program for patients with high-complexity diabetes led to a significant and sustained reduction in glycated hemoglobin and hypoglycemic episodes, as well as in the rate of emergency room visits and hospitalizations. These results encourage the adoption of this technology in patients who do not achieve metabolic control with optimal management of diabetes.

## Introduction

### Background

An estimated 537 million people worldwide have diabetes, and it is projected that by 2045, more than 783 million people will have the disease (a prevalence of 12.2%) [1]. The complexity of this condition and its complications has led to a growing burden in health care systems. For example, the management of people with type 1 diabetes (T1D) or beta-cell failure has been challenging due to the complete lack of insulin reserve, leading to hypo- and hyperglycemia and high glucose fluctuations. To ensure adequate care for this condition, patients require education on diabetes, dietary advice, knowledge on counting carbohydrates, and the application of multiple daily injection (MDI) of insulin, including dosage adjustment. In some situations, the use of sensor-augmented insulin pump (SAP) therapy, which combines continuous subcutaneous insulin infusion (CSII) and continuous glucose monitoring (CGM), is fundamental to achieving glucose targets.

The current American Diabetes Association (ADA) and International Society for Pediatric and Adolescent Diabetes (ISPAD) guidelines recommend the therapeutic goal of glycated hemoglobin (HbA_1c_) <7% in most patients (or <7.5% or <8% depending on the risk of hypoglycemia), access to technology, and the ability to manage their condition [2,3]. In addition, there is widespread evidence of the limitations of using HbA_1c_ as the only guide for managing diabetes. The lack of information regarding intra- and interdaily glycemic excursions and the acute complications derived from hyper- and hypoglycemia are some of the reasons for considering other tools for managing diabetes [4-6].

With recent advances over the last few years, such as better safety profiles in insulin pharmacokinetics and new diabetes devices, the risk of hypoglycemia has been reduced, and patients have been able to achieve better HbA_1c_ goals [7-12]. The benefits of adjusting treatment based on continuous glucose monitoring metrics (time in range, time below range, time above range, and coefficient of variation) have led to their widespread use and resulted in better metabolic control in patients and the reduction of hypoglycemic episodes [13-17]. However, the quality of diabetes programs is vitally important for both the care and management of these patients, to achieve adequate metabolic control, and to prevent micro- and macrovascular complications, as well as hypoglycemic episodes, emergency room visits, and hospitalizations.

Although there is evidence of the benefit of CSII therapy for glycemic control [18-20] and the reduction of hypoglycemia [14,21], other studies have not demonstrated the usefulness of this type of therapy [22,23], and there is scant evidence of its long-term benefit in reducing emergency room visits and hospitalizations [24].

The objective of our study was to evaluate the long-term effects of SAP therapy in patients at a specialized diabetes care center who were using this type of technology. We present a retrospective longitudinal study with a median 4-year follow-up.

## Methods

### Design and Participants

This was an analytical, retrospective observational study designed to evaluate the long-term effect of the use of SAP on clinical outcomes and metabolic control after the admission of people with diabetes to a specialized care program using this type of technology. The included patients were affiliated to a health care insurance company in Bogotá, Colombia (Compensar Entidad Promota de salud).

The inclusion criteria were being aged 18 years or older; having a diagnosis of type 1, 2, or another type of diabetes; and being on a basal-bolus insulin regimen. All patients were referred to our center due to nonoptimal metabolic control (HbA_1c_ >7%) or frequent hypoglycemic episodes (<70 mg/dl), defined as >4 hypoglycemic events per week or one episode of severe hypoglycemia (needing a third party for correction assistance) during the last year [2,10,25,26] despite optimal treatment and diabetes self-management education and support (DSMES), which was defined as the adequate use of insulin self-titration, frequent self-monitoring of blood glucose (SMBG; at least 4 times a day), management of hypoglycemic episodes, and education in carbohydrate counting based on international and local guidelines for T1D management [27-29].

Prior to SAP therapy, all patients in our center were using insulin analogues (long-acting insulin glargine 100 units/mL and fast-acting insulin, ie, lispro, aspart, or glulisine); after 2017, they used second-generation basal insulin (ie, insulin glargine 300 units/mL or insulin degludec 100 units/mL). The patients used SAP therapy from the time of their admission to the program, following the consensus statement of the insulin pump management task force [30,31]. The data were collected from that point and patients were recruited from January 2013 until November 2020. No patients were excluded, given the specific focus of the program in which they were participating.

The primary outcomes of the study were to evaluate clinical and glycemic control, including changes in glycemic control, the proportion of patients who achieved an HbA_1c_ less than 7%, the number of severe hypoglycemia (SH) episodes and nonsevere hypoglycemia (NSH) episodes, and the number of hospitalizations or emergency room visits prior to beginning the program and during follow-up in the insulin pump program.

All study participants used an insulin pump (Paradigm VEO, MiniMed 640G or 670G, Medtronic Inc) and real-time CGM (Enlite or Guardian Link 3, Medtronic Inc). On initiation of SAP, all participants were trained by the Medtronic team. During the first 3 days of SAP, they received advice on diabetes and nutrition and intensive classes on how to manage the SAP technology. They were subsequently contacted over the next 3 days to verify the correct use of SAP, its drawbacks, and how to solve them. They were encouraged to perform SMBG at least 6 times per day, sensor calibration 3 to 4 times per day, and infusion set changes (the reservoir, catheter and cannula) every 3 days. Based on medical criteria, the patients were re-educated in carbohydrate counting and management of hypo- and hyperglycemic episodes and encouraged to follow the correct use of SAP throughout follow-up. Initially, the follow-up visits were done face-to-face monthly or every 2 months. Owing to the COVID-19 pandemic, consultations were conducted by means of telephone. The data were obtained from a chart review, the insurance company’s database for their affiliated hospitals, and direct patient surveys. Adherence to treatment and the measurement of variables related to insulin pump use were reviewed using the CareLink Medtronic system software. We used the Gold scale as an assessment tool for hypoglycemia awareness, with lower values indicating a greater perception of hypoglycemia and higher values reflecting a lack of perception [32].

To minimize bias, the data were independently reviewed by one of the authors to assess biologically implausible or missing data.

### Statistical Analysis

The categorical variables were expressed as absolute and relative frequencies. The Kolmogorov-Smirnov test was used to evaluate the normality of numerical variables. Parametric data are expressed as the mean (SD), while nonparametric data are reported as the median (IQR).

Changes in variables over time were evaluated with a 2-tailed Student *t* test for paired data or the McNemar test for categorical variables. The Wilcoxon test was used for paired data and the Friedman test was used for nonparametric data.

### Ethics Approval

The study was approved by the local ethics committee in Bogotá, Columbia (Clinical Committee, Cardioinfantil Foundation, Cardiology Institute), on February 11, 2021 (04-2021).

## Results

### Patient Characteristics

We analyzed data from 74 patients who used SAP between January 2013 and August 2020. During the follow-up period, 1 patient was lost in the first year due to changes in their residential address and health care provider, making it unfeasible to continue their evaluation. Another person left the program after 4 years for the same reason, but their data were included in the analysis.

The median age was 36 (IQR 27-46) years. The median BMI was 24.3 (IQR 22.7-27.2) kg/m^2^. Of the total population, 41 (55%) were female and most had a diagnosis of T1D (n=71; 95%), with a median of 20 (IQR 14-33) years since diagnosis. The median number of years of follow-up after starting to use SAP was 4 (IQR 2-7) years. Altogether, 85% (63/74) of the patients had glycemia above the optimal target, with a median HbA_1c_ of 8.35% (IQR 7.3%-9.8%). The baseline demographic and clinical characteristics are shown in Table 1. In our country, SAPs are currently the only device provided by health care insurance. CSII alone has never been a treatment option in Colombia. The CGM Freestyle Libre flash glucose monitoring system became available in 2019.

The hospitalization and emergency room visit rates prior to SAP therapy were 0.5 (IQR 0.5-1.0) events per patient-year and 1.0 (IQR 0.5-2.0) events per patient-year, respectively. All patients had a history of hypoglycemic episodes with an NSH rate of 20 (IQR 11-35) events per patient-year and an SH rate of 1.5 (IQ 1-6) events per patient-year, with a Gold score of 4 (IQR 2-4); the mean total daily insulin dose was 52.5 (SD 21.9) international units (IU) prior to treatment.

**Table 1:** Baseline patient characteristics (N=74).

Characteristics			Values
Male, n (%)			32 (45)
Female, n (%)			42 (55)
Age (years), median (IQR)			36 (27-46)
BMI (kg/m^2^), median (IQR)			24.3 (22.7-27.2)
Type 1 diabetes, n (%)			71 (96)
Type 2 diabetes, n (%)			2 (3)
Diabetes, other, n (%)			1 (1)
Duration of diabetes (years), median (IQR)			20 (14-33)
Basal bolus, n (%)			74 (100)
Total daily insulin doses (international units), mean (SD)			52.5 (21.9)
**Type of pump^a^, n (%)**			
	Paradigm VEO	6 (8)	
	MiniMed 640	40 (54)	
	MiniMed 670	28 (38)	
Glycated hemoglobin (%), mean (SD)			8.8 (2.7)
Glycated hemoglobin (%), median (IQR)			8.35 (7.3-9.8)
Gold score, median (IQR)			4 (2-4)
Gold score ≥4, n (%)			44 (56)
Nonsevere hypoglycemia episodes, EPY^b^ (IQR)			20 (11-35)
Severe hypoglycemia episodes, EPY (IQR)			1.5 (1-6)
Hospitalization rate, EPY (IQR)			0.5 (0.5-1.0)
Emergency room visit rate, EPY (IQR)			1.0 (0.5-2.0)

^a^Type of pump initiated at the start of follow-up.

^b^EPY: events per patient-year.

In the first year, the median percentage sensor use, the mean number of SMBG measurements per day and the mean number of calibration readings per day were 90% (IQR 95%-90%), 5.83 (SD 1.48), and 5.22 (SD 2.96), respectively. At the end of the follow-up period, these values were 89.5% (IQR 80%-92%), 4.14 (SD 0.89), and 4.43 (SD 1.07), respectively. More detail is given in Table 2.

**Table 2:** Variables associated with sensor-augmented insulin pump technology during different stages of the follow-up period.

	First year	Second year	Third year	Final follow-up
Sensor use, % (IQR)	90 (95-90)	80.5 (65.5-90)	89.5 (80-94)	89.5 (80-92)
Number of self monitoring of blood glucose measurements per day, mean (SD)	5.83 (1.48)	5.53 (1.45)	5.03 (1.39)	4.14 (0.89)
Number of calibration readings per day, mean (SD)	5.22 (2.96)	5.03 (1.59)	4.80 (1.51)	4.43 (1.07)

### Metabolic Control During Follow-Up

**Figure 1:**
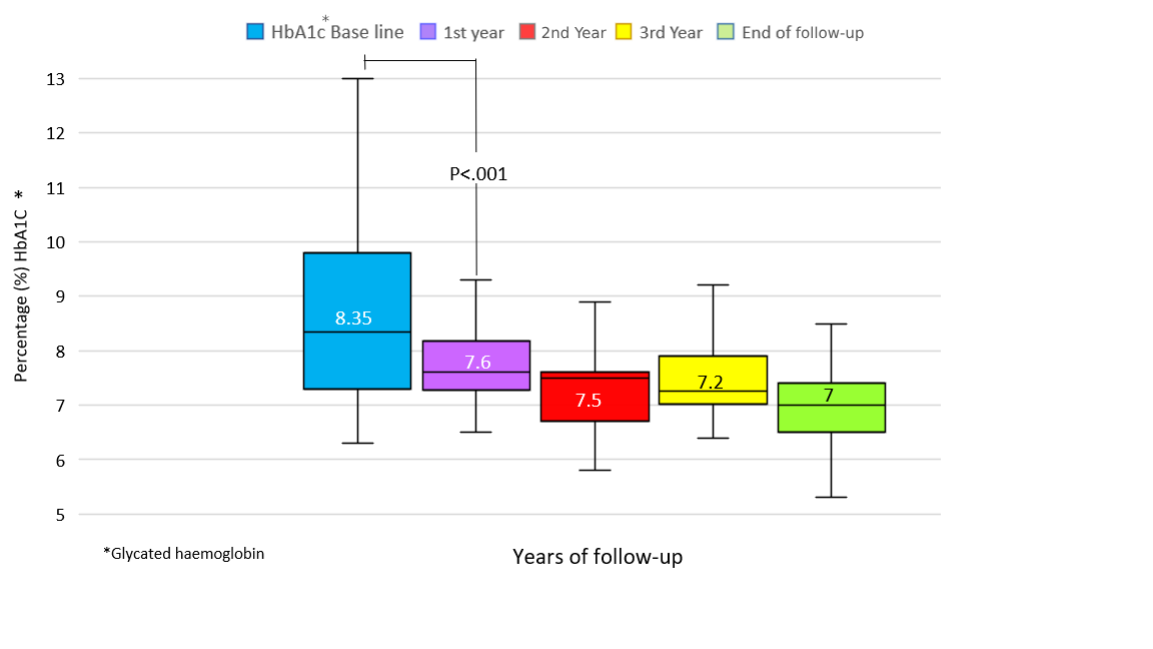
HbA_1c_ during follow-up. HbA_1c_: glycated hemoglobin.

A significant improvement in glucose control was observed at the final follow-up, with the median HbA_1c_ decreasing to 7% compared with the baseline of 8.35% (*P*<.001). The percentage of patients with an HbA_1c_ less than 7% prior to treatment was 15% (11/74), and this increased significantly to 41% (30/74) of patients at the end of follow-up (*P*<.001; Figure 1).

During the follow-up period, a noteworthy reduction in HbA_1c_ levels was observed within the first year, decreasing from 8.35% (IQR 7.3%-9.8%) to 7.6% (IQR 7.3%-8.1%; *P*<.001). In the second year, HbA_1c_ levels remained consistent at 7.5% (IQR 6.7%-7.7%), and in the third year and the final follow-up, stabilized at 7.25% (IQR 7%-7.9%) and 7% (IQR 6.5%-7.4%), respectively. These findings indicate an initial improvement followed by a sustained stabilization in HbA_1c_ levels among the patients under study.

At the end of follow-up, the median time in range (TIR) for a blood glucose level 70 to 180 mg/dL was 75.5% (IQR 70%-80.5%), with 21% (IQR 15%-29%) time above 180 mg/dL and 3% (IQR 1%-4%) time below 70 mg/dL.

### Hypoglycemic Episodes

There was a statistically significant decrease in both the rate of NSH episodes and the percentage of patients with at least one episode in the last year, falling from 20 (IQR 11-35) episodes per patient-year to 4 (IQR 2-7) episodes per patient-year (*P*<.001) and from 96% (71/74) to 84% (62/74; *P*=.01), respectively (odds ratio [OR] 0.91, 95% CI 0.083-0.99).

Severe hypoglycemia, expressed in rates and percentage of episodes in the last year, decreased from 1.5 (IQR 1-6) episodes per patient-year to 0.5 (IQR 0.31-0.5) episodes per patient-year (*P*=.14), and from 28.3% to 14.3% (*P*=.14), respectively, but without statistical significance.

An assessment using the Gold scale was made at the beginning of SAP therapy and at the end of follow-up. The scale showed a reduction from a score of 4 (IQR 2-4) at baseline to 2 (IQR 1-3) at the end of follow-up (*P*<.001). Likewise, the percentage of patients with a score of 4 or higher prior to using this technology decreased from 60% (44/74) to 41% (30/74; *P*<.001).

### Emergency Room Visits and Hospitalizations

Significant differences were found before and after the use of SAP therapy in terms of a reduced number of hospitalizations, with an initial rate of 0.5 (IQR 0.5-1) events per person year decreasing to 0.26 (IQR 0.16-0.667) events per patient-year (*P=*.004). The percentage of patients requiring hospitalization (during the last 2 years) was 50% (36/72) prior to and 14% (10/72) after beginning SAP therapy (OR 0.23, 95% CI 0.001-0.58; *P*<.001).

Moreover, prior to beginning SAP therapy, 58% (42/73) of the patients had to be admitted to the emergency room, but at the end of the study, only 6% (4/62) of them had to be admitted (OR 0.11, 95% CI 0.01-0.83; *P*<.001). Emergency room visits fell from 1 (IQR 0.5-2.0) event per person year to 0.25 (IQR 0.16-0.6) events per person year (*P*<.001; Table 3; Multimedia Appendix 1).

**Table 3:** Follow-up data on the clinical outcomes.

Outcomes	Patients at baseline, n/N (%)	Patients at final follow-up, n/N (%)	*P* value
At least one severe hypoglycemia episode	21/74 (28)	10/70 (14)	.14
At least one nonsevere hypoglycemia episode	71/74 (96)	62/74 (84)	.01
Gold score ≥4	44/74 (60)	30/74 (40)	<.001
Required emergency room visit	42/73 (58)	4/62 (6)	<.001
Required hospitalization	36/72 (50)	10/72 (14)	<.001

## Discussion

### Principal Findings

The most relevant result of this study is the long-term (4-year) benefit we observed; there was a reduction in the number of hospitalizations and emergency room visits, in addition to better metabolic control, with the use of SAP therapy, which combines CSII and real-time CGM (rt-CGM). Many publications [19,20,24,33-35] have shown that SAP therapy has clinical and glycemic benefits in patients not controlled with a basal-bolus regimen. Previous studies, such as Gómez et al [21], have shown HbA_1c_ reductions with SAP therapy from 8.8% (SD 1.9%) to 7.5% (SD 1%) at 5 months (mean difference –1.3%, 95% CI –1.09 to –1.5; *P*<.001) and 7.1% (SD 0.8%; mean difference –1.7%, 95% CI –1.59 to –1.9; *P*<.001) after 47 months of follow-up. Likewise, the incidence of SH decreased significantly, from 66.6% to 2.7% (*P*<.001). In addition to HbA_1c_ reduction (from 8.7%, SD 1.7% to 7.4%, SD 0.8%; *P*<.05), Ramirez-Rincon et al [24] found a decline in hospitalizations, from 16.5% to 6% (*P*<.05), as well as a reduction in the incidence of SH, from 32% to 7.1%, at 1 year of follow-up. Our results are similar to those of the abovementioned studies. This confirms the utility of SAP technology in these high-complexity treatment groups. In Colombia, some health care programs perform follow-up monthly or every 3 months with an interdisciplinary team (medical and administrative support) that resolves clinical or administrative issues that might hamper adherence and glycemic control.

However, some studies have shown no benefit for metabolic control with the use of this type of technology. Blair et al [23] found no HbA_1c_ reduction or cost-effectiveness in using CSII compared with MDI (CSII: 7.72%, 95% CI 7.5%-7.94%; MDI: 7.5%, 95% CI 7.28%-7.72%). However, they evaluated results from patients aged from 7 months to 15 years. The outcomes were examined in patients with a de novo T1D diagnosis and analyzed within the first year of the disease. This protocol made it difficult to determine any benefit or difference between therapies due to complex glycemic control and known limitations in the pediatric population [36]. The glycemic and pathophysiological behavior of T1D in this age group, especially in the first year after diagnosis [37,38], may have masked the differences that might otherwise be seen in patients with a medium- to long-term duration of the disease. Bolli et al [22] found no differences between the use of MDI or CSII. The mean HbA_1c_ reduction was similar in both groups: CSII was –0.7% (SD 0.7%) and MDI was –0.6% (SD 0.8%), with an adjusted difference of 0.1% (95% CI 0.5%-0.3%). However, the patients had previously used neutral protamine Hagedorn (NPH) insulin and were randomized to a glargine insulin regimen or CSII. The design of this study limited the ability to find differences between groups, as long-acting insulin should be the standard treatment today, not a comparative alternative to CSII. The indication to initiate CSII should be in patients with untargeted glycemic control or persistent hypoglycemic events after using a basal-bolus regime with second-generation and rapid-acting insulin [30,31,39].

It should be noted that only a small (but ever-growing) group of subjects with T1D, T2D, and other types of diabetes will be able to access SAP technology due to the increasing use of CGM (with intermittent scanning or real-time monitoring) as a standard of care, with encouraging outcomes in glycemic goals and avoiding hypoglycemic episodes [7,8,40-44]. Furthermore, the economic burden of these technologies is a barrier in low-income countries; however, we think the costs will probably decrease as the technology becomes more available. Even our study demonstrates the utility and probable cost-effectiveness of the use of these technologies [45-47].

In our study, the patients had been diagnosed with T1D for an average of 20 years and had nongoal glycemic control with MDI despite having complete diabetes training, including the techniques for applying and self-titrating insulin, carbohydrate counting, managing hypoglycemia, and using second-generation insulin analogues, as recommended by T1D International and local management guidelines [27-29]. This is vitally important because in our population, SAPs are used as a step-up treatment only when the metabolic control goals are not met despite interdisciplinary and specialized management and not as an alternative treatment in patients who will potentially be controlled through optimized management with education and training in disease management.

One of the advantages of our study is that the majority of the patients used recent insulin pump models, which in other publications have been shown to be beneficial in reducing hypoglycemic episodes and HbA_1c_ [48,49]. The study by Bolli et al [22] was performed using the MiniMed 508 model, which did not have technologies such as the Bolus Wizard. The latter is useful for estimating the bolus dose using a calculation of the insulin-to-carbohydrate ratio, the insulin sensitivity factor, the target blood glucose, and active insulin. The most recent devices allow more stringent targets to be pursued, reducing the risk of hypoglycemia and the coefficient of variation [50].

Throughout the follow-up period, remarkable adherence to the therapy was recorded, with an average sensor use time exceeding 80%. This high adherence was attained through regular medical consultations and follow-ups, which were conducted at least 6 times per year for all patients, while promoting proper sensor use for as long as possible along with calibration and blood glucose measurement.

There was a small difference in the number of blood glucose measurements and calibrations when comparing the first year of follow-up to the subsequent years of follow-up, likely due to the use of new devices such as the MiniMed 670 pump, which requires fewer calibrations and has improved precision. Additionally, some level of fatigue or sense of security may have arisen from the prolonged use of these devices.

The most significant improvement in HbA_1c_ levels occurred during the first year of therapy, with a reduction from 8.35 (IQR 7.3-9.8) to 7.6 (IQR 7.3-8.1; *P*<.001). This improvement progressed gradually, reaching a median of 7.0 (IQR 6.5-7.4), which can also be attributed to technological advancements during the follow-up period.

Strict and frequent follow-up among this young population, along with consistent and adequate adherence to the therapy, allowed for high percentages of sensor use, SMBG measurements, and calibrations. These factors are reflected in our results.

### Limitations

The main limitation of this study is its retrospective character. It is a common situation in the analysis of retrospective cohorts that there is a loss of some data in the clinical history records. Some data were taken from the chart review and the insurance company’s database and others from patient surveys, which may have led to various types of bias. We performed a comprehensive review of the data and chose the worst-case scenarios.

Another limitation of our study is the lack of a control group (without SAP therapy). However, given the type of population to which we had access in this program, it was not possible to include patients without this technology and carry out long-term follow-up.

Moreover, the results may have been influenced by the trial design. The switching of the previous diabetes management regime with MDI plus SMBG to SAP is a significant step that entails a probable benefit in all outcomes, as was found in this trial. Nowadays, use of CGM is growing as a diabetes standard of care. Recently, much evidence has been published showing that CGM reduces hypoglycemic events and leads to lower HbA_1c_ with increases in TIR. In Colombia, intermittently scanned CGM (is-CGM; the FreeStyle Libre system) was the first device, approved in 2019, and its use is increasing rapidly. In our opinion, this technology promises to have clinical benefits like those demonstrated in this trial, but nevertheless this needs to be confirmed.

Finally, one interesting question is the future of CGM versus SAP as a tool for diabetes management. Choudhary et al [51] compared is-CGM and an advanced hybrid closed loop (AHCL) system. The latter showed an additional benefit in HbA_1c_ reduction (AHCL: –1.54%; is-CGM: –0.20%), resulting in a treatment effect of −1.42% (95% CI −1.74% to −1.10%; *P*<.001). Thus, new technologies such as AHCL can provide effective therapy and have advantages for the treatment of this complex disease.

### Conclusion

This study is the first to evaluate the safety, as well as the clinical and glucose benefits, of using SAP therapy in a population with T1D, with real-life data and long-term follow-up. The use of this technology for an average of 4 years led to a significant HbA_1c_ reduction, achievement of HbA_1c_ goals, and a lower number of NSH episodes, emergency room visits, and hospitalizations. These results should encourage the adoption of this technology in patients who do not achieve metabolic control with optimal care for T1D. It should be noted that its efficacy requires a multidisciplinary team with experience in the use of this technology and close patient support. Finally, we recommend carrying out experimental studies to compare this technology with other therapies.

## Appendix

Multimedia Appendix 1 Follow-up of the clinical outcomes.

## Abbreviations

ADA: American Diabetes Association
AHCL: advanced hybrid closed loop system
CGM: continuous glucose monitoring
CSII: continuous subcutaneous insulin infusion
DSMES: diabetes self-management education and support
HbA_1c_: glycated hemoglobin
is-CGM: intermittently scanned continuous glucose monitoring
ISPAD: International Society for Pediatric and Adolescent Diabetes
IU: international units
MDI: multiple daily injection
NPH: neutral protamine Hagedorn
NSH: nonsevere hypoglycemia
OR: odds ratio
rt-CGM: real-time continuous glucose monitoring
SAP: sensor-augmented insulin pump
SH: severe hypoglycemia
SMBG: self-monitoring of blood glucose
T1D: type 1 diabetes
T2D: type 2 diabetes
TIR: time in range
